# Lemon flavonoids nutraceutical (Eriomin®) attenuates prediabetes intestinal dysbiosis: A double‐blind randomized controlled trial

**DOI:** 10.1002/fsn3.3654

**Published:** 2023-09-19

**Authors:** Fernanda M. M. Ramos, Carolina B. Ribeiro, Thais B. Cesar, Dragan Milenkovic, Lucélia Cabral, Melline F. Noronha, Katia Sivieri

**Affiliations:** ^1^ Graduate Program in Food, Nutrition and Food Engineering Sao Paulo State University (UNESP) Araraquara Brazil; ^2^ Department of Nutrition University of California Davis Davis California USA; ^3^ Institute of Biosciences, Depart of General and Applied Biology São Paulo State University (UNESP) Rio Claro Brazil; ^4^ Research Informatics Core, Research Resource Center University of Illinois at Chicago Chicago Illinois USA

**Keywords:** clinical trial, eriocitrin nutraceutical, Eriomin®, intestinal dysbiosis, lemon flavonoid, microbiota (16S rRNA), pre‐diabetes

## Abstract

Eriocitrin (eriodictyol 7‐*O*‐β‐rutinoside), a citrus flavonoid from lemon juice and peel, reduces hyperglycemia and improves diabetes‐related biomarkers in prediabetes patients. Eriocitrin is first metabolized by gut microbiota, producing energy for gut cells and short chain fatty acids that play a relevant role in glycemic control. The aim of this study was to assess the effect of Eriomin®, a nutraceutical composed of 70% eriocitrin, 5% hesperidin, and 4% naringin, on the microbiota of prediabetic patients. Patients were randomly divided into two groups and received unlabeled capsules of Eriomin® (200 mg/day) or placebo during 12 weeks. After treatment with the nutraceutical, it was a 6% decrease of hyperglycemia and 22% increase of GLP‐1 blood levels of (*p* < .05). The profile of intestinal microorganisms, obtained by 16S rRNA sequencing of the patients' feces extract, showed changes in microbiota composition, such as lower growth of Firmicutes and less abundance of the *Lachnospiraceae* family. The family *Ruminococcaceae* increased and *Blautia* genus reduced with Eriomin® supplementation. In additional, *Blautia* was positively correlated with hyperglycemia reduction. In conclusion, the nutraceutical Eriomin® moderately reduced the growth of microorganisms associated with intestinal dysbiosis and increased the abundance of beneficial bacteria. Changes promoted mainly by the flavonoid eriocitrin in the microbiota were related to a lower glycemic level and increased production of GLP‐1 in patients with prediabetes.

## Graphical abstract

Patients with prediabetes treated with a lemon flavonoid nutraceutical (Eriomin®) had 6% reduction in hyperglycemia and 22% increase in GLP‐1 blood levels. Intestinal microorganisms showed lower Firmicutes growth and less *Lachnospiraceae* family abundance. There was also an increase in the *Ruminococcaceae* family and a decrease in the *Blautia* genus, which was positively correlated with the reduction in hyperglycemia. In conclusion, the nutraceutical Eriomin® moderately reduced the growth of microorganisms associated with intestinal dysbiosis and increased the abundance of beneficial bacteria. Heatmap of correlation analysis between gut microbiota and glucose, after 12 weeks of Eriomin® treatment.

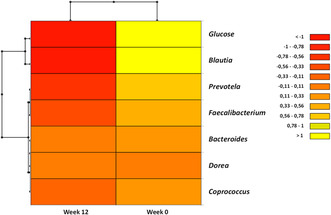

## 1 INTRODUCTION

Metabolic disorders in adults and the elderly often signal the onset of chronic degenerative diseases, with a great impact on the health and longevity of affected individuals. For example, elevated blood glucose, dyslipidemia, and prehypertension are frequent and measurable signs that precede the development of type 2 diabetes, atherosclerosis, and cardiovascular disease (Beulens et al., 2019). Prediabetes can be detected by a slight increase in fasting blood glucose of 5.6–6.9 mmol/L, as well as glucose intolerance (7.8–11 mmol/L), followed or not by an increase in glycated hemoglobin (HbA1c >5.7–6.4%) (American Diabetes Association Professional Practice Committee et al., 2022). In addition, other metabolic changes, such as excess visceral fat, chronic low‐grade systemic inflammation, and disruption of healthy gut microbiota, known as dysbiosis, constitute primary causes of degenerative diseases (Lloyd‐Price et al., 2016).

Microbiota dysbiosis reflects changes in the resistance, resilience, stability, and diversity of intestinal microorganisms, as well as quantitative and qualitative reduction in the abundance of short‐chain fatty acids (SCFAs) produced by gut bacteria (Iebba et al., 2016). Associated with this imbalance, the fragility of the intestinal barrier associated with fewer tight junctions increases the permeability and translocation of lipopolysaccharides (LPS) and Gram‐negative bacterial wall fragments, which reach the bloodstream causing metabolic endotoxemia (Cani, 2018; Zhang et al., 2013). This event stimulates the secretion of proinflammatory cytokines and activates Toll‐like receptor 4 (TLR‐4) and nuclear factor kappa B (NF‐κB) in epithelial cells, triggering low‐grade systemic inflammation, common in obese animals and humans (Gomes et al., 2017). In turn, inflammation activates macrophages infiltration in adipose tissue, liver, and kidneys, stimulating the production of tumor necrosis factor α (TNF‐α), interleukins (such as IL‐6), adipokines (such as leptin and resistin), and C‐reaction protein (CRP), causing insulin resistance and leading to prediabetes, among other metabolic disorders (Boutagy et al., 2016; Zhou et al., 2019).

Reversal of prediabetic intestinal dysbiosis may be a strategy to combat the development of type 2 diabetes mellitus (Cani, 2018). One attempt would be to modulate the gut microbiota through supplementation with phenolics, which are found in a wide variety of foods such as fruits, vegetables, herbs, seeds, and cereals, as well as in beverages such as coffee, tea, cocoa, and wine (Kasprzak‐Drozd et al., 2021). Among them, citrus‐derived flavonoids such as hesperidin, naringenin, and eriocitrin appear to benefit the health of the microbiota (Amiot et al., 2016; Ávila‐Gálvez et al., 2021).

Eriocitrin (eriodictyol 7‐*O*‐β‐rutinoside), a citrus flavonoid found primarily in lemon peel, is recognized for its anti‐inflammatory and antioxidant properties, and it has the potential to be used in diabetes therapy (Miyake et al., 1998). After its ingestion, most of the eriocitrin passes intact through the small intestine and is metabolized in the large intestine by the gut microbiota (Ávila‐Gálvez et al., 2021). We recently tested the efficacy of the citrus flavonoid eriocitrin, in reversing hyperglycemia in patients treated with the nutraceutical Eriomin® (Ribeiro et al., 2019), composed mainly of eriocitrin (70%), hesperidin, naringin, and didymin (20%), and fiber plant material (10%). The results showed a significant reduction in blood glucose associated with an increase in glucagon‐like peptide‐1 (GLP‐1) and a reduction in inflammatory biomarkers. Although an improvement of glycemic profile and a reversion of prediabetes were for 24% of patients with Eriomin®, its impact on the gut microbiota has not yet been studied. Thus, the aim of this study was to evaluate the potential of Eriomin® to modulate the intestinal microbiota and its relationship with the improvement of blood glucose in prediabetic individuals.

## 2 MATERIALS AND METHODS

### 2.1 Participants

Individuals of both sexes, aged 30–69 years, identified with prediabetes, were eligible to participate in the study. They presented at least one of the following inclusion criteria: (1) fasting blood glucose of 5.6–6.9 mmol/L, (2) glucose intolerance, as assessed by glucose tolerance test (7.8–11 mmol/L); (3) glycated hemoglobin (HBA1C) of 5.7–6.4% (American Diabetes Association Professional Practice Committee et al., 2022). However, individuals under the use of hypoglycemic drugs, weight loss drugs, antibiotics (within the last 3 months), laxatives, and/or dietary supplements (vitamins, minerals, and bioflavonoids) were excluded. In addition, individuals under intense physical training (more than 10 h/week) or with chronic diseases, such as type 2 diabetes, liver disease, renal syndrome, cardiovascular disease, polycystic ovary syndrome, or thyroid dysfunction, were excluded. Those with gastrointestinal diseases such as malabsorption syndrome, chronic inflammatory bowel disease, colorectal cancer, celiac disease, diverticulitis, and Crohn's disease, which affect the gut microbiota, were also excluded. Smokers, individuals with a history of drug or alcoholism, and pregnant women were also unable to participate.

Sixty‐two eligible individuals were selected through medical records from the public health system of Araraquara SP Brazil. They were contacted by phone by the study personnel to explain the objectives of the study, as well as obtain acceptance to voluntarily participate. Forty‐five volunteers met the inclusion/exclusion criteria and were invited to complete a clinical data questionnaire and to have blood samples withdrawn for basal tests. Of these, 29 completed the study, 16 women and 13 men, aged 50 ± 10 years.

The experimental protocol was approved by the Ethics Committee of the Pharmacy School, UNESP (CAEE: 67610217.6.0000.5426) and registered at ClinicalTrials.gov (NCT03925909). The procedures performed followed the ethical guidelines of the National Health Council (Res. 466/12) and the Declaration of Helsinki (1964), and all participants voluntarily signed an informed consent form before starting the study.

### 2.2 Study design

**FIGURE 1:**
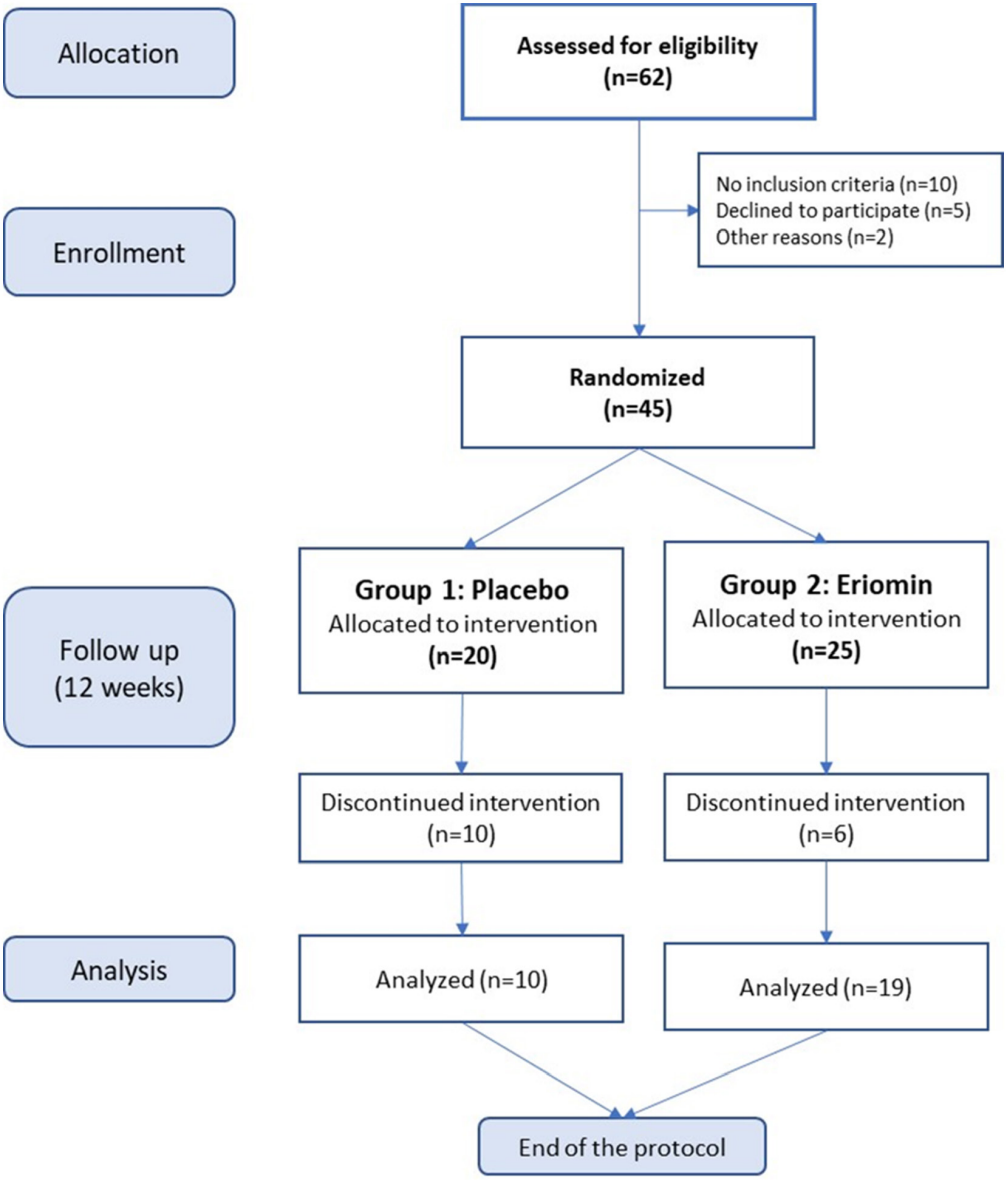
Experimental design of a 12‐week, double‐blind, randomized, placebo‐controlled, two‐arm parallel study. Of the 62 eligible candidates, 45 subjects were selected and separated randomly into two groups: Placebo (*n* = 20) and Eriomin® (*n* = 25). For 12 weeks they received a daily dose of the designated supplement, and after subtracting the dropouts, 29 subjects participated in the final analysis of the study.

A double‐blind, randomized, placebo‐controlled study began with 45 individuals randomly divided into two groups: Eriomin® and placebo. Each patient, out of a total of 45, was numbered according to their casual entry into the trial, from 1 to 45 subject. Then, following a random list of 45 numbers, the first 25 were chosen to be the patients in the experimental group. The remaining 20 random numbers were assigned to patients in the placebo group. All subjects were treated with a 200 mg/day of Eriomin® (*n* = 25) or the placebo (*n* = 20). They received instructions to maintain their usual diet during the intervention and minimize the intake of citrus foods and flavonoid‐rich beverages, such as coffee, tea, cocoa, fruit juice, and wine. Eriomin® and placebo capsules in unlabeled vials were provided to the participants every 2 weeks. The volunteers took one capsule per day with water after dinner for 12 weeks. Sixteen subjects were excluded during the experimental period due to moving away (*n* = 2), incorrect or irregular use of capsules (*n* = 10), use of antibiotics due to an unrelated problem (*n* = 1), and intentional weight loss (*n* = 3). Twenty‐nine subjects who followed the experimental protocol recommendations were included in the final analysis. The chosen dose of Eriomin® (200 mg/day) was based on our previous study (Ribeiro et al., 2019), which showed no difference in lowering blood glucose after 200, 400, or 800 mg of Eriomin® for 12 weeks (Ribeiro et al., 2019). The experimental design is shown on Figure 1.

### 2.3 Supplement preparation

The intervention product was Eriomin®, a supplement of citrus flavonoids supplied by Ingredients by Nature TM, Montclair, CA. Purity, as determined by HPLC, was 70% eriocitrin, 5% hesperetin, 4% naringin, 1% didymin, and 20% of fiber material from epidermis and periderm cell‐wall, composed by suberin, cutin, lignin, pectin, and cellulose. The dose of 200 mg of active ingredients per capsule was equal to 140 mg of eriocitrin, plus 10 mg of hesperidin, plus 8 mg of naringin, and plus 2 mg of didymin. The placebo was composed of pure microcrystalline corn starch. Placebo and Eriomin® were encapsulated in tablets of the same size, shape, and color, by a registered pharmacist, and the appearance was identical between for both placebo and the active supplement.

### 2.4 Sampling and storage of blood and feces

The 12 h fasting blood samples were collected at the beginning of the first week and at the end of the 12th week of the protocol, and the blood serum samples were kept at −80°C. The collection and storage were performed at the Clinical Analysis Laboratory São Lucas, Araraquara‐SP. Fecal samples were self‐collected by the volunteers in a commercial sterile container, provided by the laboratory, at the beginning of the first week and at the end of the 12th week. The samples, delivered to the laboratory within 24 h of collection, were homogenized, stored in sterile plastic tubes (10 mL), and kept at −80°C in a deep freezer (Haier Biomedical) until analysis.

### 2.5 Microbiota

DNA extraction from stool samples was performed using the “PowerLyzer @PowerSoil DNA Isolation Kit” (Qiagen). Primers 319F/806R were used to amplify the V3–V4 region of the 16S rRNA. In step 1, the forward and reverse primers contained a sequence of Illumina tags, a variable‐length spacer, a binding sequence, and the target sequence 16S to increase diversity and improve the quality of sequencing and execution. Each 25 μL PCR reaction contained a Kapa2G Robust Hot Start Polymerase unit (Kapa Biosystems), 1.5 mmol/L MgCl_2_, 0.2 mmol/L dNTP mixture, 0.2 μmol/L of each primer, and 1 μL of DNA for each sample. In step 2, each sample was encoded with a unique back‐and‐forth barcode combination with an Illumina P5 adapter string, a unique 8 nt barcode, a partial match string of the lead adapter used in step 1, and reverse primers with an Illumina P7 adapter. The PCR reaction in step 2 contained a final concentration of 0.2 μmol/L of each primer with a unique barcode and 1 μL of the product of the PCR reaction in step 1. The final product was quantified on the Qubit instrument using the kit of Qubit Broad Range DNA (Invitrogen), and the individual amplicons were pooled in equal concentrations. The pooled library was cleaned using Ampure XP beads (Beckman Coulter), and the band of interest was subjected to isolation by 1.5% gel electrophoresis (Sage Science). The library was quantified via qPCR followed by 300 bp paired‐end sequencing using an Illumina MiSeq instrument at the Genome Center DNA Technologies Core at UC Davis.

Demultiplexing the Raw FASTQ files and adjusting the sequence adapter were performed using dbcAmplicons v. 0.8.5 (http://github.com/msettles/dbcAmplicons). Non‐immersed direct and reverse readings were imported into QIIME2 version 2017.12 (https://qiime2.org), and the sequence variants were determined following the DADA2 analysis pipeline. To classify the sequences according to their taxonomic information, the q2‐feature‐classifier plugin was used according to the VSEARCH alignment method (Gurevich et al., 2013) with the SILVA v132 database (Rognes et al., 2016) and with a sequence identity of 99%. The 16S rRNA gene sequencing analyses were carried in R (Lahti & Eckermann, 2019; McMurdie & Holmes, 2013) Studio, version 3.2.4 (R Core Team, 2018) using the Phyloseq and Microbiome packages.

### 2.6 Biochemical analysis

Fasting glucose, oral glucose tolerance test, HbA1c, insulin, total cholesterol, HDL‐cholesterol, and triglycerides were measured using commercial kits (Labtest). The homeostasis model assessment of insulin resistance (HOMA‐IR) was estimated using a cutoff point ≥2.71 (Matthews et al., 1985). The following metabolic and inflammatory markers were analyzed by Luminex Milliplex® (RP3X Scientific): glucagon‐1‐like peptide (GLP‐1), glucagon, C‐peptide, adiponectin, tumor necrosis factor alpha (TNF‐α), interleukin 6 (IL‐6), and ultrasensitive C‐reactive protein (CRP‐us). Lipid peroxidation was evaluated using the TBARS assay (Yagi, 1998), and the total antioxidant capacity was evaluated using the ABTS^+^ radical assay (Re et al., 1999). The following hepatic and renal biomarkers were evaluated using commercial kits (Labtest): aspartate transaminase (AST), alanine transaminase (ALT), alkaline phosphatase, gamma‐glutamyl transferase (γGT), and creatinine.

### 2.7 Biometric measurements

The following anthropometric parameters were evaluated using a high‐frequency tetrapolar bioimpedance equipment (Inbody 720®): weight (kg), height (m), body mass index (BMI) (kg/m^2^), lean mass (kg), fat mass (kg), body fat (%), and visceral fat area (cm^2^). Participants were instructed to fast for 2 h before bioimpedance, not drink alcohol or caffeine in the last 24 h, wear light clothes, urinate 20 min before the test, remove any type of metallic adornment, and not practice physical activity in the last 24 h. Waist circumference was measured at the midpoint between the lower border of the last rib and the top of the iliac crest (World Health Organization, n.d.). Blood pressure was measured using a digital monitor (ReliOn, HEM‐741 CREL).

### 2.8 Food and energy consumption

Participants were instructed to maintain their usual diet and physical activity throughout the 12 weeks of the experiment. They were also asked to complete a 3‐day food diary (two weekdays and one weekend day) during the first and last week of the study. Participants recorded in detail everything they ate or drank, including the culinary preparations (fried, boiled, baked, etc.), as well as the added salt, sugar, seasonings, and sauces. Once completed, a nutritionist reviewed the diary with each patient to add any missing information.

Mean daily food intake was estimated on the basis of the 3 days recorded. The analysis of energy, macronutrient, and micronutrient intake was performed using the DietBox® software, according to the Brazilian Food Composition Table (NEPA‐UNICAMP, 2011). The quantification of the total energy expenditure (TEE) was estimated on the basis of the dietary recommended intakes (DRIs) regarding the overweight and obese population, considering each individual's sex, age, height, weight, and level of physical activity. Then, the average energy consumption was compared with the average TEE (DRI ref).

### 2.9 Conformity assessment, adverse effects, and safety of the supplement

In the biweekly consultation with a nutritionist, participants were asked to return unused capsules from the previous period, and they received a new batch for the next 15 days. At each visit, participants were asked about adverse events or discomfort caused by the nutraceutical intervention. Adherence to the treatment was assessed by counting capsules untaken and the absence of undesirable effects. Participants who consumed more than 90% of the capsules and who provided and completed all assessments were considered to have good adherence to treatment and were included in the statistical analysis. The others were removed from the final analyses.

### 2.10 Randomization and sample size estimation

The number of participants for this clinical trial was based on the mean change in fasting blood glucose after 12 weeks of treatment with the nutraceutical Eriomin® (Cesar et al., 2022). In the present study, patients with prediabetes were randomized into two groups in a 1:1 ratio. Randomization sequence and sample size, with a statistical significance level of 5% and test power of 80%, were estimated using Sigma Stat, v.3.11, Systat Software, USA. The minimum sample size was estimated at 10 patients per group; and considering a hypothetical dropout rate of 15%, the protocol started with at least 12 subjects per intervention group, and at the end had at least 10 patients per group.

### 2.11 Statistical analysis

The data are presented as the mean ± SD. The normality of each set of data was evaluated using the Kolmogorov–Smirnov or Shapiro–Wilk test. Two‐way ANOVA on ranks, followed by post hoc analysis (Holm–Sidak method), was used to detect changes in biochemical, clinical, and dietetic factors.

Unilateral ANOVA (normal data distribution) and Kruskal–Wallis (non‐normal data distribution) were used to identify differences between microbiota parameters at the baseline and at the end experiment. Statistical analysis was performed using SigmaStat 3.0 or BioEstat 5.0 software, and statistical significance considered was *p* ≤ .05.

Pearson correlation analysis was used to evaluate the correlation between gut microbiota and biochemical variables (fasting glucose) in the beginning and after 12 weeks of Eriomin® treatment. A correlation network plot was generated, and correlation magnitudes >1.0 (strong co‐abundance relationships) and <−1.0 (strong co‐exclusion relationships) were plotted. Visualization of the network was performed by XLSTAT software.

## 3 RESULTS

Adult men (*n* = 21) and women (*n* = 24), aged between 32 and 69 years, selected according to the prediabetes criteria from ADA (American Diabetes Association Professional Practice Committee et al., 2022), participated in this study. Two registered dietitians followed them before and during the clinical trial (12 weeks), and 29 subjects completed all protocol steps and were included in the study. Table 1 summarizes the baseline parameters of the selected subjects. According to these parameters, on average, patients had fasting blood glucose ≥100 mg/dL, HbA1c ≥5.7%, HOMA‐IR ≥2.71, body mass index ≥35 kg/m^2^, and systolic blood pressure ≥ 120 mmHg. Together, these parameters indicated a prediabetes condition with insulin resistance, obesity, prehypertension, and a high risk of developing type 2 diabetes (American Diabetes Association Professional Practice Committee et al., 2022).

**TABLE 1:** Baseline characteristics of prediabetic female and male volunteers before treatment with Eriomin® or Placebo for 12 weeks.

Baseline parameters	Female	Male	Total	Prediabetes cutoff
	*n* = 16	*n* = 13	*n* = 29	
Age, years	48.3 ± 10.7	51.9 ± 7.8	49.9 ± 9.6	≥35
Glucose, mg/dL	100 ± 9	112 ± 9	105 ± 11	≥100–125
Insulin, μU/mL	21.6 ± 7.5	15.0 ± 8.5	18.6 ± 8.5	–
Insulin Resustance (HOMA‐IR) a	5.2 ± 1.5	4.1 ± 2.3	4.7 ± 2.0	≥2.71
Glycated Hemoglobin (HbA1c), %	5.8 ± 0.3	6.1 ± 0.6	6.0 ± 0.5	≥5.7%
Triglycerides, mg/dL	154 ± 54	180 ± 91	166 ± 73	≥250
Total Cholesterol, mg/dL	198 ± 48	198 ± 42	198 ± 45	≥240
HDL‐cholesterol, mg/dL	46 ± 7	41 ± 8	44 ± 8	<35
Weight, kg	91.5 ± 17.6	96.0 ± 20,6	93.7 ± 18.9	–
Body Mass Index, kg/m^2^	34.9 ± 6.4	35.1 ± 6.7	33.4 ± 6.7	≥25
Waist Circumference, cm	114 ± 24	108 ± 16	111 ± 21	≥90
Systolic Blood Pressure, mmHg	119 ± 10	122 ± 13	121 ± 12	≥120
Diastolic Blood Pressure, mmHg	75.5 ± 8.2	79.3 ± 11.6	76.0 ± 10.4	≥80

*Note*: Data presented as mean ± SD.

**FIGURE 2:**
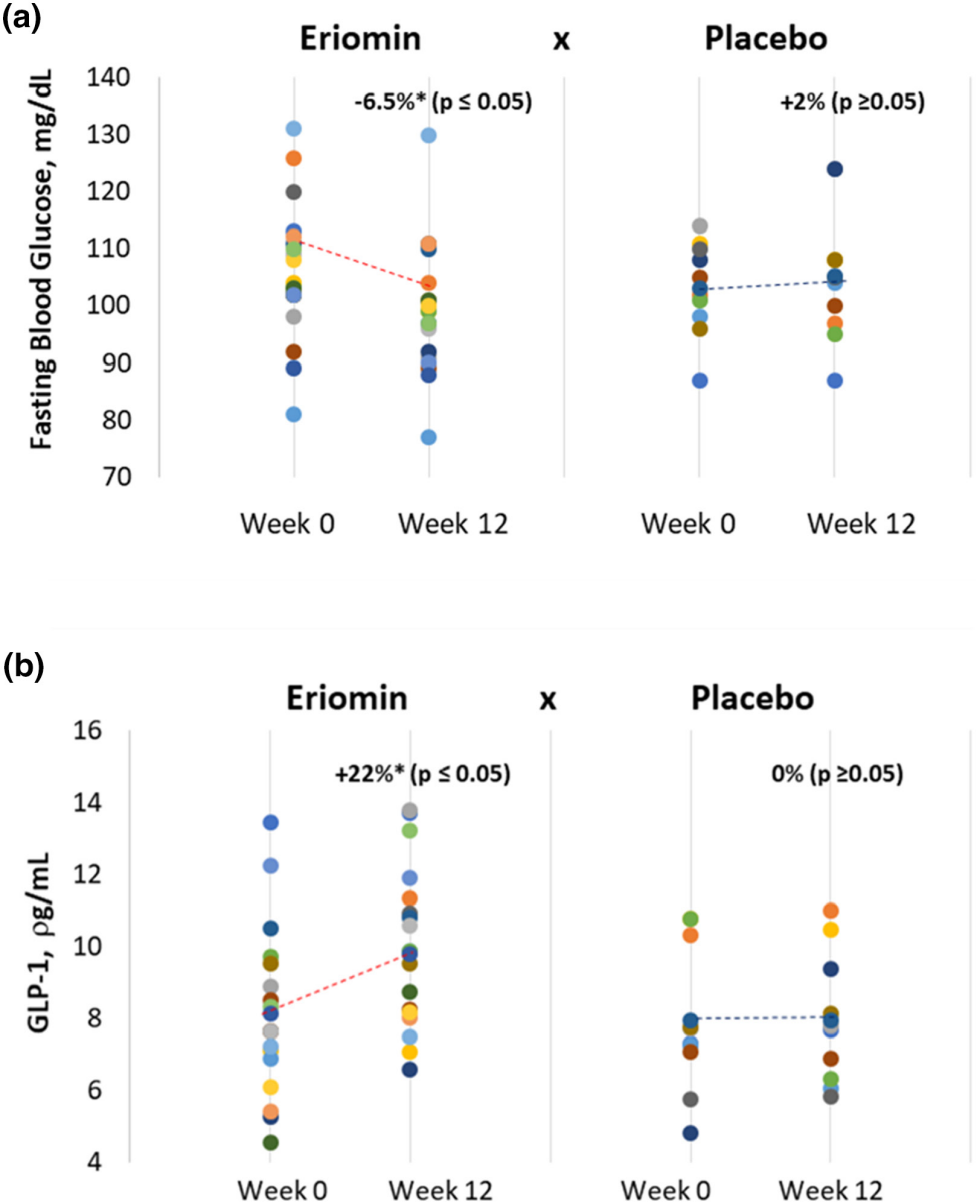
Course of (a) blood serum glucose (mg/dL) and (b) GLP‐1 over 12 weeks of treatment (Week 0 and Week 12) with 200 mg/day of Eriomin versus Placebo in prediabetes patients. Circular points represent individual measurements of Glucose (a) and GLP‐1 (b). Dash line represents the average trend line over time.

Blood serum biomarkers before and after Eriomin® and placebo supplementation for 12 weeks are shown in Table 2. As expected, for prediabetic subjects, fasting glucose, HOMA‐IR, and HbA1c are above the reference values, but after Eriomin® treatment, statistical analysis revealed a 6.5% decrease in fasting blood glucose after 12 weeks (*p* ≤ .05), while patients who received the placebo remained with unchanged levels over time (Table 2 and Figure 2a). Conversely, blood serum GLP‐1 levels in patients treated with Eriomin® significantly increased by 22% (*p* ≤ .05), whereas the placebo intervention revealed no change (*p* ≥ .05; Table 2 and Figure 2b). Mean blood levels of total cholesterol and LDL‐cholesterol were borderline high, indicating a high risk of developing atherosclerosis, but with some protection provided by HDL‐cholesterol above 35 mg/dL (NHLBI, 2013).

**TABLE 2:** Blood serum biomarkers of prediabetic volunteers treated with Eriomin® or Placebo daily supplements for 12 weeks.

Variables	Placebo		Eriomin®	
	0 week	12°week	0 week	12°week
Glucose, mg/dL	103 ± 8	105 ± 12	106 ± 12	99.0* ± 11.0
Insulin, μU/mL	19.5 ± 8.2	18.4 ± 9.9	18.1 ± 8.9	17.7 ± 8.3
HOMA‐IR	4.97 ± 2.34	4.99 ± 3.47	4.53 ± 1.78	4.37 ± 1.88
HbA1c, %	5.86 ± 0.42	5.79 ± 0.47	6.01 ± 0.53	5.87 ± 0.48
Total Cholesterol, mg/dL	222 ± 55	222 ± 45	185 ± 33	185 ± 39
LDL‐Cholesterol, mg/dL	140 ± 40	141 ± 34	102 ± 31	101 ± 35
HDL‐Cholesterol, mg/dL	44.6 ± 10.2	43.7 ± 11.1	44.5 ± 8.5	43.8 ± 7.0
Triglycerides, mg/dL	167 ± 75	189 ± 75	165 ± 74	168 ± 65
Glucagon‐like peptide‐1 (GLP1) ρg/mL	7.96 ± 2.06	7.97 ± 1.82	8.15 ± 2.3	10.0* ± 2.2
Glucagon, ρg/mL	147 ± 13	142 ± 18	144 ± 17	130 ± 11
Adiponectin, μg /mL	18.5 ± 5.21	19.6 ± 4.8	18.1 ± 9.4	20.4 ± 6.4
C Peptide, ρg/mL	1779 ± 483	1743 ± 532	2290 ± 861	2009 ± 671
hsCRP, mg/dL	0.39 ± 0.25	0.32 ± 0.15	0.37 ± 0.22	0.33 ± 0.18
IL‐6, ρg/mL	7.62 ± 5.34	7.61 ± 6.14	5.86 ± 2.50	4.87 ± 1.74
TNF‐α, ρg/mL	5.45 ± 1.35	5.18 ± 1.75	5.66 ± 1.36	5.03 ± 1.51
Antioxidant capacity, μM	1.88 ± 0.04	1.89 ± 0.03	1.86 ± 0.10	1.90 ± 0.03
Lipid peroxidation (MDA) mM	2.25 ± 1.22	2.05 ± 1.21	1.60 ± 0.76	1.49 ± 0.68
Alkaline phosphatase (ALP) U/L	67.3 ± 12.1	67.3 ± 18.0	57.6 ± 13.2	57.3 ± 14.5
γ‐glutamyl transferase (γGT), U/L	57.2 ± 34.8	68.3* ±37.0	38.8 ± 32.4	39.2 ± 29.0
Aspartate transaminase (AST), U/L	26.6^b^ ± 8.2	29.9^b^ ± 8.0	19.4^a^ ± 4.0	21.1^a^ ± 4.5
Alanine transaminase (ALT), U/L	35.3^b^ ± 17.4	40.1^b^ ± 14.9	20.2^a^ ± 6.3	22.4^a^ ± 8.3
Urea, mg/dL	31.5 ± 8.7	32.5 ± 9.4	34.2 ± 15.0	32.3 ± 9.0
Creatinine, mg/dL	0.90 ± 0.15	0.92 ± 0.23	0.85 ± 0.29	0.86 ± 0.21

*Note*: Data are presented as mean ± SD. Statistical analysis: Two‐way ANOVA on Ranks, followed by Holm–Sidak to detect changes in intragroup (week 0 vs. week 12) and intergroups (Placebo vs. Eriomin).

* Statistical difference significant intragroup (week 0 vs. week 12) (*p* ≤ .05).

^a ≠ b^ Statistical difference significant intergroups (Eriomin vs. Placebo) (*p* ≤ .05).

Neither Eriomin® nor placebo promoted significant changes in glucagon, C‐peptide, adiponectin, hsCRP, IL‐6, TNF‐α, antioxidant capacity, or lipid peroxidation levels (Table 2). Liver enzymes, ALP, AST, and ALT, showed higher values in the placebo group from the beginning toward the end of the experiment, without any changes in between. In contrast, γ‐GT increased after 12 weeks in the placebo group, but not with Eriomin® supplement (Table 2).

Statistical analysis of biometric, hemodynamic, and dietary parameters of prediabetic volunteers undergoing treatment with Eriomin® or the placebo for 12 weeks showed no change with the supplements after the treatment period (Table 3).

**TABLE 3:** Clinical and dietetic parameters of prediabetic volunteers submitted to Eriomin® or Placebo daily supplements for 12 weeks.

Variables	Placebo		Eriomin®	
	0 week	12°week	0 week	12°week
*Biometric*				
Systolic BP, mmHg	125 ± 10	124 ± 11	102 ± 25	102 ± 26
Diastolic BP, mmHg	78.0 ± 7.9	74.0 ± 7.7	79.0 ± 9.4	77.0 ± 9.5
Body weight, kg	103 ± 17	104 ± 17	102 ± 25	102 ± 26
BMI, kg/m^2^	34.5 ± 6.5	34.5 ± 6.7	34.5 ± 7.1	34.5 ± 7.3
Lean mass, kg	34.5 ± 7.3	34.7 ± 7.4	34.9 ± 6.7	34.9 ± 7.3
Fat mass, kg	37.7 ± 8.9	38.3 ± 15.6	40.1 ± 17.8	39.3 ± 18.6
Waist/Hip	1.05 ± 0.10	1.05 ± 0.09	1.06 ± 0.08	1.06 ± 0.09
*Nutritional*				
Energy, kcal/day	3538 ± 932	3500 ± 927	3180 ± 855	3249 ± 1006
*(TEE*, *kcal/day)*	*(2453 ± 320)*	*(2383 ± 392)*	*(2318 ± 356)*	*(2419 ± 306)*
Carbohydrates, g/day	380 ± 82	415 ± 126	374 ± 119	360 ± 98
*(AMDR = 45–65%TEE)*	*(44 ± 8%)*	*(48 ± 10%)*	*(47 ± 6%)*	*(46 ± 9%)*
Protein, g/day	143 ± 52	138 ± 46	135 ± 45	135 ± 45
*(AMDR = 10–35%TEE)*	*(16 ± 3%)*	*(16 ± 4%)*	*(17 ± 3%)*	*(17 ± 5%)*
Lipids	162 ± 66	145 ± 38	129 ± 35	142 ± 76
*(AMDR = 20–35% TEE)*	*(40 ± 8%)*	*(38 ± 11%)*	*(36 ± 6%)*	*(42 ± 25%)*
Saturated Fat Acids	37 ± 16	49 ± 12	40 ± 10	44 ± 20
*(AHA ≤7% TEE)*	*(13 ± 3%)*	*(13 ± 4%)*	*(11 ± 2%)*	*(13 ± 7%)*
Fibers, g/day	17.8 ± 3.3	19.5 ± 3.9	19.6 ± 3.0	19.4 ± 3.8
*(AI = 25 g/day)*	*Below*	*Below*	*Below*	*Below*
Cholesterol, mg/day	566 ± 188	554 ± 117	553 ± 156	504 ± 118
*(AHA ≤ 200 mg/day)*	*Above*	*Above*	*Above*	*Above*

*Note*: Data presented as mean ± SD. The values in *(italic)* represent % of macronutrient energy (kcal/day) regarding the Total Energy Expenditure (TEE). No statistic differences detected intragroup (week 0 vs. 12) or intergroups (Eriomin vs. Placebo) (*p* ≥ .05).

Abbreviations: AHA, American Heart Association; AI, Adequate Intake; AMDR, acceptable macronutrient distribution ranges; TEE, total energy expenditure (kcal/day).

The unbalanced and hypercaloric dietary pattern is noticeable by the high energy consumption (>3000 kcal), above the Total Energy Expenditure (TEE = 2400 kcal/day) for these obese and sedentary individuals (Table 3). In addition, the distribution of macronutrients in kcal (Acceptable Macronutrient Distribution Ranges, AMDR) showed high consumption of total lipids (~40%) and saturated fats (~13%), exceeding the recommendation for individuals with cardiovascular risk (25% and 7%, respectively). High consumption of dietary fats indicated a high‐fat diet, associated with high consumption of cholesterol (>500 mg/day). In addition, there was a low consumption of dietary fiber (17 g/day), whose recommendation by American Heart Association (AHA) for adults is ≥30 g/day (Cesar et al., 2022).

### 3.1 Effects of Eriomin® on gut microbiome

A total of 5,187,450 high‐quality readings were obtained from microbiota samples collected at the beginning and end of the placebo and Eriomin® treatments. After normalizing the data, 868,564,000 sequences were produced. Alpha diversity showed no significant changes in OTU richness, Chao1, Simpson, and Shannon indices between the Eriomin® and placebo groups (Table 4).

**TABLE 4:** Microbiota alpha diversity indexes (Chao1, Shannon, and Simpson) obtained for all fecal samples before and after Placebo and Eriomin daily supplements.

	Placebo		Eriomin®		
	0 week	12°week	0 week	12°week	*p‐*Value
Chao1	1919 ± 225	1713 ± 232	1745 ± 29	1702 ± 222	.416
Shannon	5.9 ± 0.6	5.7 ± 0.6	5.7 ± 0.1	5.7 ± 0.3	.587
Simpson	0.9 ± 0.05	0.9 ± 0.03	0.9 ± 0.004	0.9 ± 0.001	.546

^a^ p‐values calculated by Kruskal‐Wallis test

Beta diversity based on PERMANOVA statistical analysis revealed significant differences in bacterial communities between 0 and 12 weeks of Eriomin® supplementation (*p* = .039), whereas the placebo group showed no statistical difference (*p* = .998; Table 5). These results indicate that the 12 weeks of Eriomin® supplementation improved the intestinal microbiota, but no change was observed in the Placebo group.

**TABLE 5:** PERMANOVA statistical analysis from beta‐diversity data using Adonis script in QIIME on week 0 and 12 of treatment (^a^ *p* ≤ .05).

Groups	*opts$category*	Df	SumOfSqs	MeanSqs	F model	R2Pr(>F)	Pr(>F)
Eriomin®	qiime.data$map	5	1.248	0.250	1.118	0.095	0.039^a^
	Residuals	53	11.829	0.223		0.904	
	Total	58	13.076			1.000	
Placebo	qiime.data$map	1	0.147	0.147	0.690	0.037	0.998
	Residuals	18	3.826	0.213		0.963	
	Total	19	3.973			1.000	

^a^ *p* ≤ .05.

**FIGURE 3:**
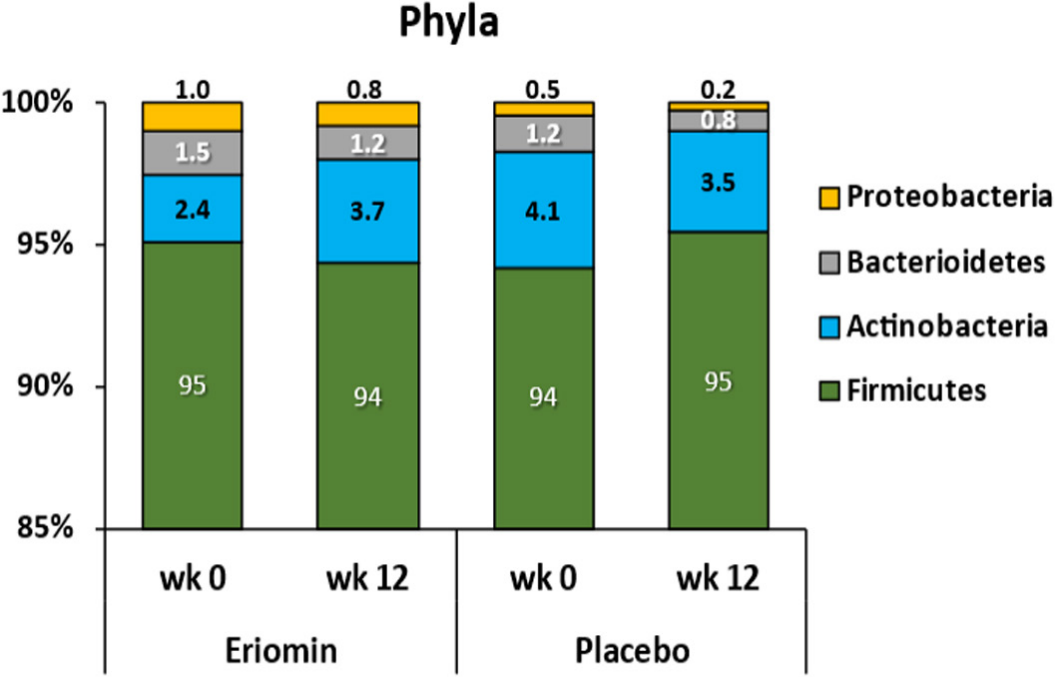
Distribution of intestinal bacterial phyla: Firmicutes, Actinobacteria, Bacteroidetes, and Proteobacteria before (week 0) and after (12 weeks) treatment with Eriomin® or placebo. Data are expressed as percentage of relative abundance by group mean in each experimental period.

Microorganism phyla distribution of prediabetic patients shown small differences between Eriomin® and placebo throughout the experimental period (Figure 3). In general, both groups showed greater abundance of the phylum *Firmicutes* (≥90%), before and after supplementation. Phylum *Actinobacteria* was the second most abundant, in average 3.5% of the total phyla, and after 12 weeks increased 1.5% with Eriomin®, but decreased 0.6% with placebo. Together, *Bacteroidetes* and *Proteobacteria* accounted for less than 3% of the total phyla abundance, and decreased slightly in both groups. This profile shows a microbiota in dysbiosis (Figure 3).

**FIGURE 4:**
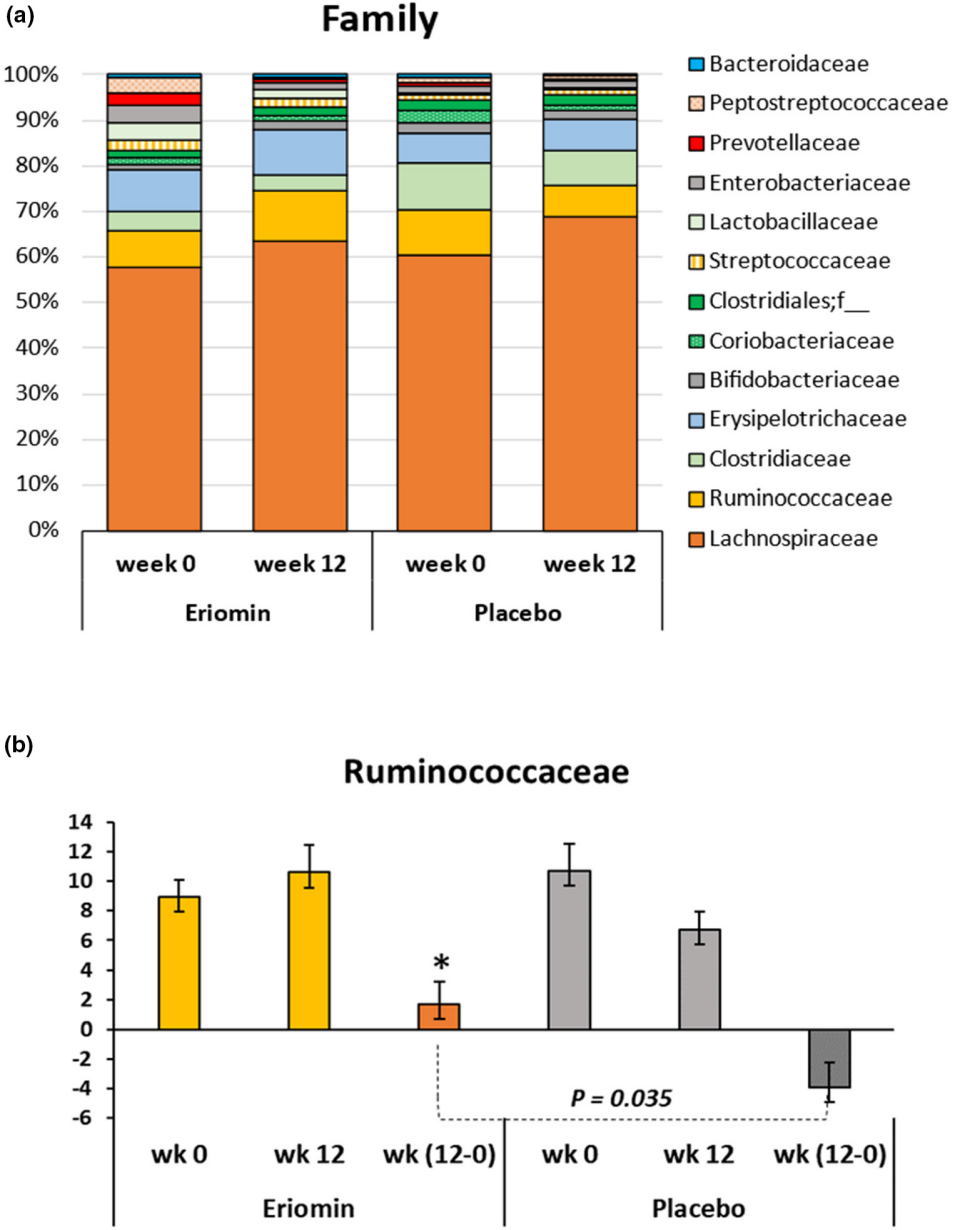
(a) Distribution of the main microbial families before (week 0) and after (week 12) treatment with Eriomin® or Placebo. Data are expressed as percentage of relative abundance by group mean (Eriomin® or Placebo) in each experimental period. (b) Changes in the abundance of the *Ruminococcaceae* family after 12 weeks of Eriomin® treatment. Values are expressed in OTU. The increment of *Ruminococcaceae* with Eriomin® versus the decrease after Placebo is statistically significant (*p* ≤ .035).

**FIGURE 5:**
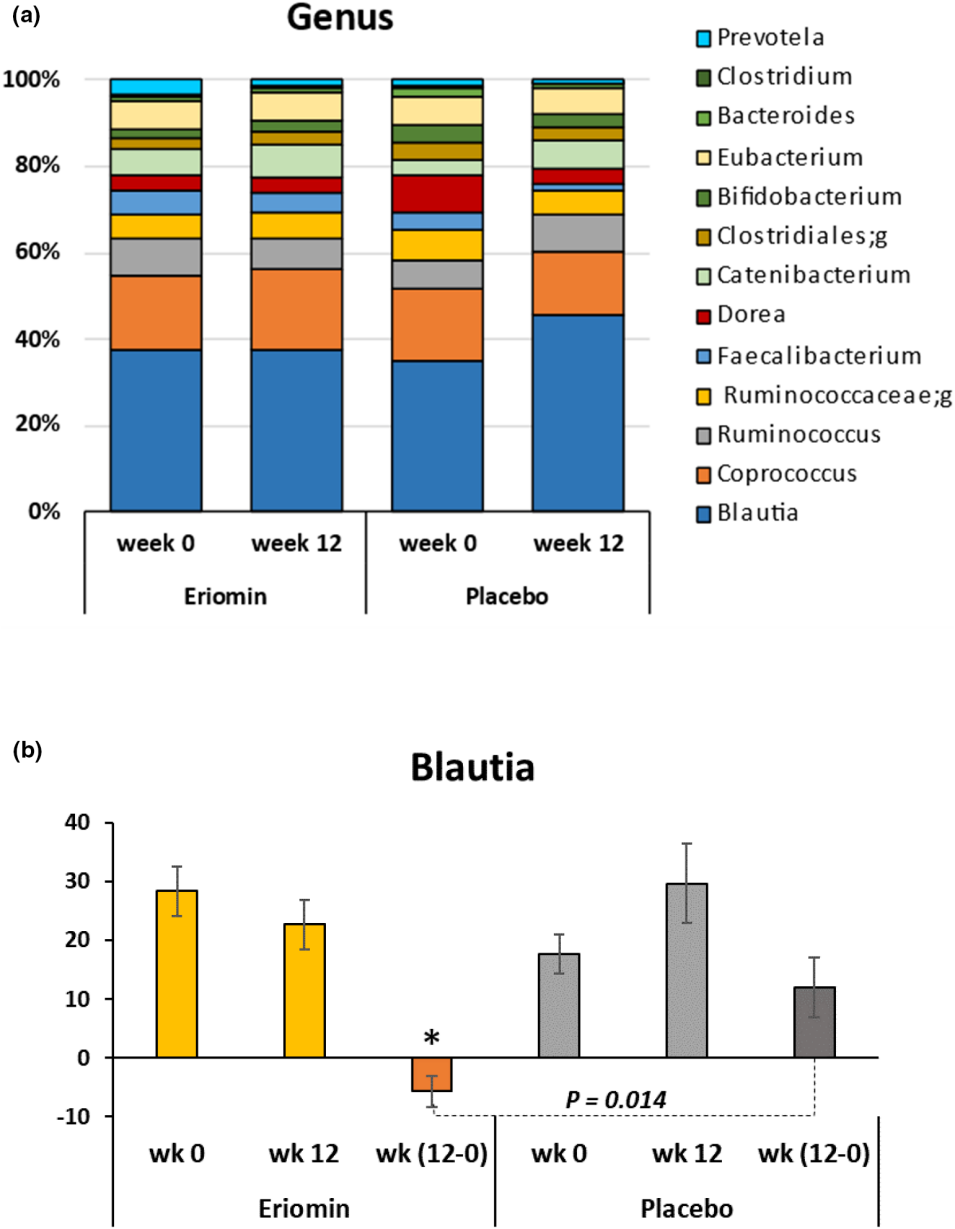
(a) Distribution of the main microbial genera before (week 0) and after (week 12) treatment with Eriomin® or Placebo. Data are expressed as percentage of relative abundance by group mean in each experimental period (week 0 and week 12). (b) Changes in the abundance of Blautia genus after 12 weeks of Eriomin® treatment. Values are expressed in OTU. The increment of Blautia with Eriomin® versus the decrease after Placebo is statistically significant (*p* ≤ .014).

**FIGURE 6:**
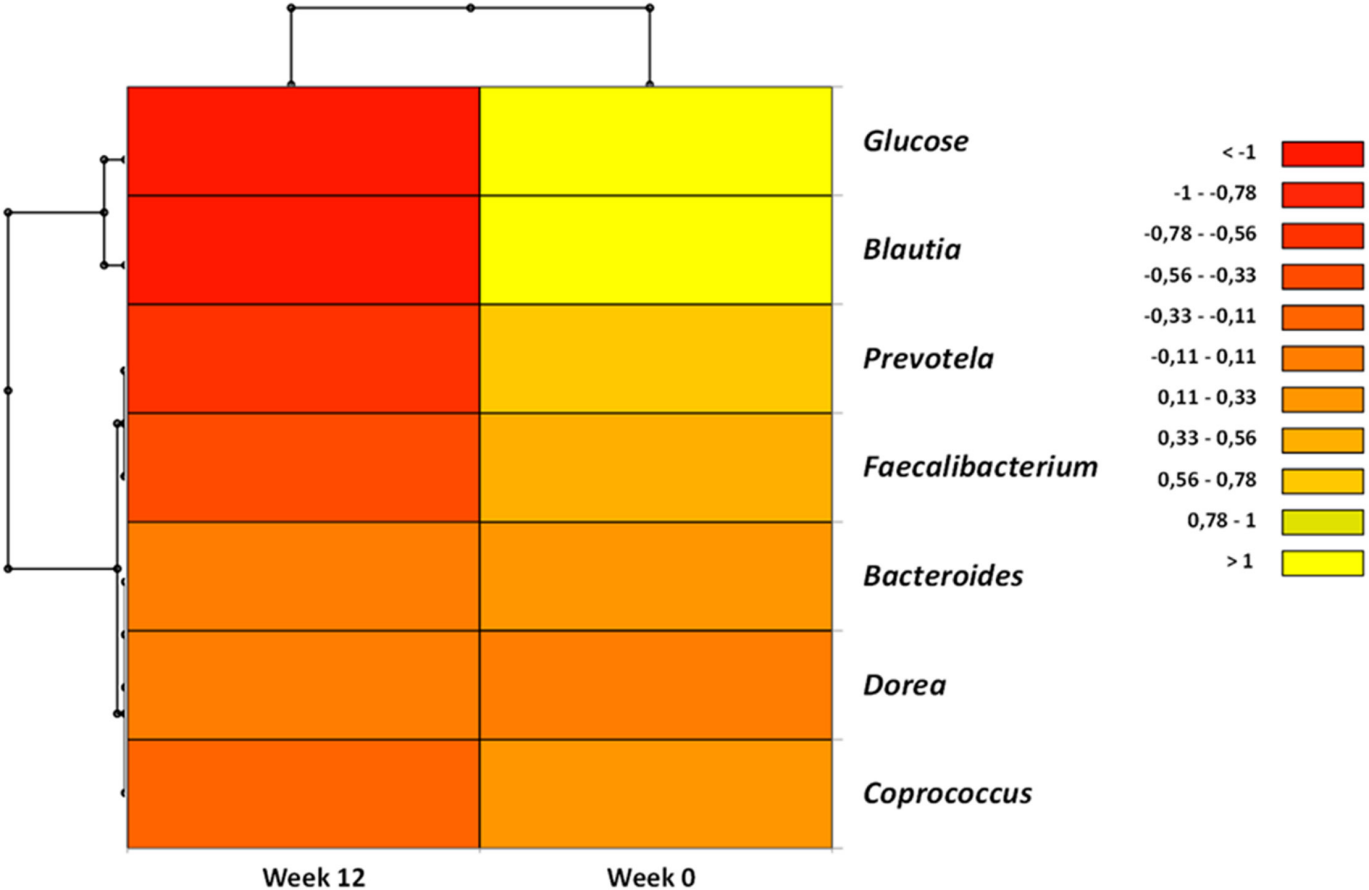
Heatmap of Pearson's correlation analysis between gut microbiota and glucose, after 0 and 12 weeks of Eriomin® treatment.

The taxonomic assignment performed at the family and genus level of the prediabetes microbiota showed similar distribution between placebo and Eriomin® (Figures 4a and 5a), with higher abundance of *Lachnospiraceae* family (>50%) and *Blautia* genus (>35%) before supplementation. *Ruminococcaceae* family increased 38% after 12 weeks of Eriomin®, in contrast with a decrease of 31% with placebo (*p* = .04; Figure 4b). At the genus level with Eriomin® treatment, it was a decreased of *Blautia* in contrast with placebo (*p* = .002), and lower levels of *Prevotella* genus, while placebo it not changed (*p* = .04; Figure 5b). The correlation analysis between blood glucose and the main genera of bacteria modulated by Eriomin® identified an interesting correlation of the decrease in Blautia and blood glucose after 12 weeks of treatment with Eriomin®, as shown in Figure 6. Therefore, to a limited extension, it was seen that Eriomin® modulated one family and two bacteria genera of the prediabetes microbiota.

## 4 DISCUSSION

Short‐term intervention of prediabetic subjects with the nutraceutical Eriomin® not only reduced hyperglycemia and increased blood GLP‐1 secretion but also had a significant effect on beta diversity in the intestinal colon, changes that were related to the improvement of prediabetic dysbiosis. This is the first time that the effect of the citrus flavonoid, eriocitrin, taken as the nutraceutical Eriomin®, has been studied on the human microbiota to treat hyperglycemia in prediabetic adults. Previous clinical studies with eriocitrin (Cesar et al., 2022; Ribeiro et al., 2019) and data from rodents (Ferreira et al., 2020; Miyake et al., 1998) showed an improvement in glucose metabolism with different doses of this compound.

Our previous clinical study (Ribeiro et al., 2019) showed the effectiveness of Eriomin® in reducing fasting glucose, glucose intolerance, insulin resistance (HOMA‐IR), glycated hemoglobin (HbA1c), glucagon, C‐peptide, HSCRP, IL‐6, and TNF‐α. Increasing doses of Eriomin® were tested (200, 400, and 800 mg/day), but the lowest dose of 200 mg/day was sufficient to achieve all critical benefits, such as an increase in GLP‐1, an improvement in the inflammatory condition and metabolic rate, and a reversal of hyperglycemia in 24% of treated patients (Ribeiro et al., 2019). Likewise, an experimental study carried out in our laboratory with rodents (Ferreira et al., 2020) showed that a lower dose of Eriocitrin (25 mg/kg) was more effective in attenuating the metabolic impairment of glucose and lipids in mice treated with an obesogenic diet than a higher dose of 100 mg/kg. Currently, the prediabetic and diabetic state has been characterized by presenting microbiota in dysbiosis, which translates into a disturbance in the abundance and variety of microorganisms in the large intestine. However, recent studies have shown that citrus‐derived flavonoids such as hesperidin, naringenin, and eriocitrin help balance the microbiota species (Ávila‐Gálvez et al., 2021). Thus, on the basis of the metabolic benefits obtained previously in prediabetic and diabetic patients treated with Eriomin® (Cesar et al., 2022; Ribeiro et al., 2019), we aimed to verify the effect of the nutraceutical Eriomin® on the intestinal dysbiosis, typical of this condition.

At the beginning of this clinical trial, all prediabetic patients had intestinal microbiota with predominance of the *Firmicutes* phylum to *Bacteroidetes* with a ratio of 63:1 and 77:1, respectively, for the Eriomin and placebo groups. The prominence of *Firmicutes* to *Bacteroidetes* typically demonstrates a microbiota in dysbiosis, common in diabetics and prediabetics, as well as in individuals with excess body weight and body fat (Zhang et al., 2013). After 12 weeks of treatment, the ratio of *Firmicutes* to *Bacteroidetes* increased by 25% with Eriomin® and 60% with the placebo, showing that Eriomin® moderated the growth of Firmicutes. The other phyla, Actinobacteria and Proteobacteria, were not modified by the treatments, and were stable at lower levels across the experiment period. The greater abundance of the phylum *Firmicutes*, with respect to the other phyla, is associated with a diet rich in carbohydrates and a high metabolic efficiency in the energy production of these strains of bacteria. Concomitantly, the high intake of energy‐rich nutrients by the host, mainly lipids, was a relevant factor in the maintenance of obesity and the presence of inflammatory markers, even after treatment with Eriomin®. About 25% of the energy consumed in excess was associated with total lipids and saturated fat in the diet. Typically, a hyperlipidemic and hypercaloric diet increases the inflammatory process, alters the expression of intestinal proteins, and weakens the selective permeability of the intestinal barrier. These events in turn lead to the translocation of cell‐wall fragments of Gram‐negative bacteria and lipopolysaccharides, which fuel low‐grade inflammation with a direct impact on blood glucose dysregulation (Ferreira et al., 2021).

Simultaneously, an abundance of 60% *Lachnospiraceae*, a representative family of the phylum *Firmicutes*, was detected in both groups at the beginning of the study. This family is associated with glycemic dysregulation, metabolic syndrome, and diet‐related obesity, as well as the prediabetic state (Lippert et al., 2017). However, Eriomin® treatment slowed the growth of *Lachnospiraceae* (2%) while placebo increased it by 11%, showing that Eriomin® attenuated the growth of this bacteria family. In contrast, the *Ruminococcaceae* family increased by 38% with Eriomin®, but decreased by 31% with the placebo. This family has been associated with the fermentation of complex carbohydrates, such as fibers and resistant starch, and polyphenols, to produce SCFA. Some SCFA, such as propionic acid, can strength the intestinal barrier and reduce systemic low‐grade inflammation associated with glucose intolerance and diabetes (Salonen et al., 2014). A recent preclinical study that evaluated the metabolism and microbiota modulation of eriocitrin showed that homoeriodictyol, one of the main metabolites of eriocitrin, was positively correlated with *Ruminococcus* (member of *Ruminococcaceae* family; Meng et al., 2023).

We also observed that the genus *Blautia* from the *Lachnospiraceae* family did not change with Eriomin® supplementation, but increased 62% under the placebo, showing a significative difference between treatments. *Blautia* is a commensal microorganism and plays a relevant role for the biotransformation of flavonoids and SCFA production, helping to maintain intestinal balance (Kim et al., 2014; Liu et al., 2021). It has been suggested that *Blautia* can regulate T cells and decrease intestinal inflammation, and has potential probiotic properties (Kim et al., 2014). Despite these relevant activities in maintaining intestinal health, the relationship of *Blautia* with metabolic diseases is still controversial (Liu et al., 2021). For example, *Blautia* is abundant in obese and prediabetic patients and contains a large amount of potentially pathogenic bacteria (Ottosson et al., 2018). Furthermore, *Blautia* is associated with high intestinal permeability, which can lead to prediabetes‐related metabolic endotoxemia (Lambeth et al., 2015). *Blautia* depletion, however, has been described in obese children with metabolic inflammation and insulin resistance (Benítez‐Páez et al., 2020). Thus, further studies are needed to identify the species, or strains of bacteria, associated with human pathologies. As Eriomin® showed relative stability in the abundance of *Blautia* in relation to the placebo group, it is suggested that the dominant species of *Blautia* in the prediabetic microbiota would be correlated with the increase in glycemia, while the arrest, or decrease, in its growth benefits insulin sensitivity and the reduction of plasma glucose, as observed in the positive correlation between the drop in glycemia and the non‐growth of Blautia in these patients. Furthermore, an excessive energy intake (>3000 kcal/day) and high consumption of fats and saturated fatty acids, combined with low intake of dietary fiber (<25 g/day) was observed for all patients. Dietary fiber intake is recognized as one of the important mechanisms for the health of the microbiota, as it provides essential nutrients for the growth of fermentable bacteria, such as *Ruminococcaceae* family, which are responsible for the production of short‐chain fatty acids (Salonen et al., 2014).

The strengths of this study were: the double‐blind and randomized experimental design, with placebo as control, in addition to the longitudinal monitoring of biochemical and metabolic parameters, and the bioinformatics of the intestinal microbiota over time as well. Among the weaknesses of this study should be mentioned the loss of participants at the end of the experiment, in addition to the self‐reported food intake during the experiment.

In summary, the nutraceutical Eriocitrin showed beneficial effects on the microbiota of prediabetic patients, such as increasing bacterial diversity and reducing the growth rate of *Firmicutes* and *Lachnospiraceae*, which are associated with glycemic dysregulation. *Blautia* genus, which is linked to inflammatory disorders and altered intestinal permeability, were also reduced with the nutraceutical treatment. Furthermore, Eriomin® increased the abundance of the *Ruminococcaceae* family, which is associated with the production of SCFA and anti‐inflammatory cytokines. Most of these effects were distinct from placebo and associated with improvement of glucose metabolism and increased levels of GLP1, which has shown to be modulated by specific strains of the gut microbiota (Tomaro‐Duchesneau et al., 2020). These results suggest that lemon flavonoid (Eriomin®) showed a sum of mild effects on the microbiota, associated with a significant improvement in the glycemic metabolism, showing a potential application in prediabetic patients. But, due to the variability of the predominant microorganisms in prediabetic and diabetic microbiota, future studies are needed to confirm the effects observed in the current study.

## AUTHOR CONTRIBUTIONS

**Fernanda M. M. Ramos:** Data curation (equal); formal analysis (equal); writing – original draft (equal). **Carolina B. Ribeiro:** Data curation (equal); formal analysis (equal). **Thais B. Cesar:** Conceptualization (equal); formal analysis (equal); funding acquisition (equal); project administration (equal); writing – original draft (equal); writing – review and editing (equal). **Dragan Milenkovic:** Formal analysis (equal); writing – review and editing (equal). **Lucelia Cabral:** Formal analysis (equal). **Melline F. Noronha:** Formal analysis (equal). **Katia Sivieri:** Conceptualization (equal); methodology (equal); supervision (equal); writing – review and editing (equal).

## FUNDING INFORMATION

This study was funded by a grant from Ingredients by Nature (Montclair, CA, USA) and supported by a Coordination for the Improvement of Education Personnel (CAPES) scholarship to FR and CR.

## CONFLICT OF INTEREST STATEMENT

The authors declare no conflicts of interest. The sponsors had no role in the design, execution, interpretation, or writing of the study.

## ETHICS STATEMENT

The experimental protocol was approved by the Ethics Committee of the Pharmacy School, UNESP (CAEE: 67610217.6.0000.5426) and registered at ClinicalTrials.gov (NCT03215043). The procedures performed followed the ethical guidelines of the National Health Council (Res. 466/12) and the Declaration of Helsinki (1964), and all participants voluntarily signed an informed consent form before starting the study.

## Data Availability

Data available on request due to privacy/ethical restrictions.
